# [Corrigendum] Repositioning of duloxetine to target pancreatic stellate cells

**DOI:** 10.3892/ol.2026.15789

**Published:** 2026-07-30

**Authors:** Akiko Sagara, Kohei Nakata, Sokichi Matsumoto, Weiyu Guan, Tomohiko Shinkawa, Chika Iwamoto, Naoki Ikenaga, Kenoki Ohuchida, Masafumi Nakamura

Oncol Lett 22: 744, 2021; DOI: 10.3892/ol.2021.13005

Subsequently to the publication of the above article, an interested reader drew to the authors’ attention that, concerning the cellular migration assay data shown in Fig. 4A on p. 7, the ‘+Duloxetine’ and ‘+PSC-SN’ data panels showed an overlapping section, such that these were apparently derived from the same original source where the results of differently performed experiments were intended to have been portrayed.

After re-examining their original data, the authors have realized that the data correctly shown for the ‘+Duloxetine’ data panel had inadvertently been duplicated as the ‘+PSC-SN’ data panel. As a result, the images originally shown for the PSC-SN migration group were incorrectly taken from the duloxetine group. After reviewing the laboratory notebooks and primary data files, the authors have identified the correct data panel for the ‘+PSC-SN’ experiment, and the revised and corrected version of Fig. 4 is shown on the next page. The authors are grateful to the Editor of *Oncology Letters* for allowing them this opportunity to publish a Corrigendum, and all the authors agree with its publication. Furthermore, the authors apologize to the readership for any inconvenience caused.

## Figures and Tables

**Figure 4.**
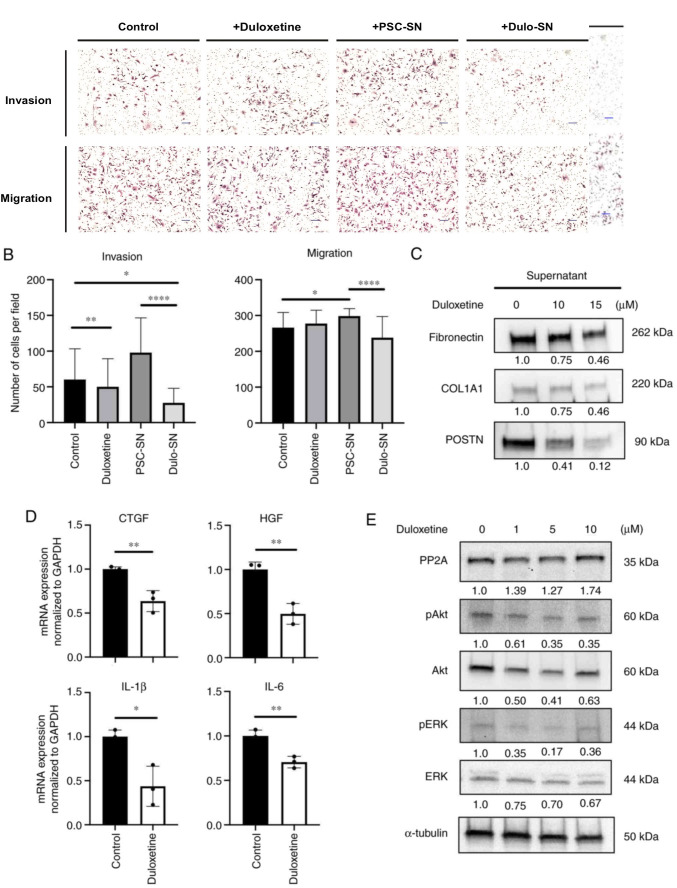
Duloxetine suppresses tumor-stromal interactions by attenuating secretomes from PSCs. (A) Representative photomicrographs of invading and migrating SUIT-2 cells in monoculture and indirect coculture with drugs or supernatants following hematoxylin and eosin staining. Magnification, ×100. Scale bar, 100 µm. (B) Graphs show the number of invading and migrating SUIT-2 cells. (C) Western blotting of extracellular matrix proteins in PSC supernatant and drug-treated PSC supernatant. (D) Relative mRNA expression of growth factors and cytokines associated with tumor-stromal interactions in PSCs treated with duloxetine. The expression levels of each gene were normalized to GAPDH. (E) Western blotting of PP2A and related proteins in whole cell lysates of PSCs treated with duloxetine at various concentrations (1, 5 and 10 µM). Values indicate densitometric ratios normalized to α-tubulin. *P<0.05, **P<0.01, ****P<0.0001 vs. control group. PSCs, pancreatic stellate cells; PSC-SN, PSC supernatant; Dulo-SN, supernatant from PSCs treated with duloxetine; PP2A, protein phosphatase 2A; COL1A1, α-1 type-1 collagen; POSTN, periostin; CTGF, connective tissue growth factor; HGF, hepatocyte growth factor; IL-1β, interleukin-1β; IL-6, interleukin-6; p, phosphorylated.

